# A Review on the Development of an Integer System Coupling Forward Osmosis Membrane and Ultrasound Waves for Water Desalination Processes

**DOI:** 10.3390/polym14132710

**Published:** 2022-07-01

**Authors:** Bara A. K. Al-Sakaji, Sameer Al-Asheh, Munjed A. Maraqa

**Affiliations:** 1 Department of Civil and Environmental Engineering, College of Engineering, United Arab Emirates University, Al-Ain P.O. Box 15551, United Arab Emirates; sakajijust@yahoo.com (B.A.K.A.-S.); m.maraqa@uaeu.ac.ae (M.A.M.); 2 Department of Chemical Engineering, College of Engineering, American University of Sharjah, Sharjah P.O. Box 2666, United Arab Emirates; 3 National Water and Energy Center, United Arab Emirates University, Al-Ain P.O. Box 1551, United Arab Emirates

**Keywords:** forward osmosis, ultrasound, water flux, draw solution, concentration polarization, fouling, scaling, seawater

## Abstract

This review considers the forward osmosis (FO) membrane process as one of the feasible solutions for water desalination. Different aspects related to the FO process are reviewed with an emphasis on ultrasound assisted FO membrane processes. The different types of membranes used in FO are also reviewed and discussed; thus, their configuration, structure and applications are considered. Coupling ultrasound with FO enhances water flux through the membrane under certain conditions. In addition, this review addresses questions related to implementation of an ultrasound/FO system for seawater desalination, such as the impact on fouling, flow configuration, and location of fouling. Finally, the mechanisms for the impact of ultrasound on FO membranes are discussed and future research directions are suggested.

## 1. Introduction

Freshwater scarcity is one of the main challenges currently faced at the global level. This is mainly due to the sharp growth in the human population and its industrial/agricultural activities [1]. Thus, current research efforts in the area of water desalination and water treatment need to address this issue in order to find appropriate solutions for the current water crisis. Seawater and brackish groundwater are the major sources of desalinated water with contributions of about 58.9% and 21.2%, respectively, with minor contributions from saline wastewater and surface water [2].

The main processes for water desalination that are mostly used worldwide are either phase change thermal driven processes, such as multiple-effect distillation (MED), multi-stage flash (MSF), and vapor compression (VC); or single-phase mass transfer driven membrane-based processes, such as reverse osmosis (RO), ultrafiltration (UF), nanofiltration (NF), and electro-dialysis (ED) [3]. Since 2010 until the end of 2019, the global desalinated water capacity increased at an average yearly growth rate of around 7%. Up to mid-February 2020, the global cumulative desalinated water production capacity reached around 114.9 million m^3^/d produced by around 21,000 projects [4]. Since 2010, membrane-based desalination techniques were the most commonly used methods in the desalination industry, where 90% of the awarded desalination contracts utilized membrane treatment systems, mainly RO systems [5]. However, most of the Middle East countries are still using thermal processes for large-scale capacity projects [5]. Thermal treatment processes are associated with high energy consumption and require costly infrastructure that is typically built adjacent to a power plant to supply the required heat source for steam generation [3].

Despite the several advantages associated with RO systems, the relatively high power demand needed by the process compared to the other treatment techniques that are used to treat low salinity feedwater, and the high tendency for membrane fouling are still challenging concerns [6]. As such, there is a need to develop an efficient desalination process in conjunction with other treatment processes, such as precipitation [7], membrane distillation (MD) [8], ultrafiltration (UF) [9], nanofiltration (NF) [10,11], and RO [12,13], which can treat high salinity water, such as seawater and brine. In order to produce high-quality water in an economic manner, the energy requirements and fouling tendency can be reduced by adopting an operating principle that does not require an external high hydraulic pressure source [14]. The forward osmosis (FO) membrane process is one feasible solution for such challenges that has been recently considered [15].

FO technology is still under development. Many research ideas and scenarios were proposed toward further development and shifting the process from bench-scale or pilot plant to reliable seawater commercialized scale applications [16,17]. A few manufacturers have succeeded, such as Modern Water plc, which installed and commissioned the world’s first commercial FO seawater desalination plant (200 m^3^/day) for treating a saline seawater feed (total dissolved solids of 55,000 mg/L) at Al-Najdah area, Sultanate of Oman [18,19]. However, the implementation of the FO has been limited due to some operational problems that prevent the process from being used properly in large-scale commercial applications. Some of these issues are related to the regeneration/separation of the draw solution, reverse solute diffusion from the draw solution side to the feed solution, or vice versa, internal/external concentration polarization (CP), and fouling problems [20,21]. These limitations have negative impact on system performance in terms of a reduction in water flux and recovery rate, which ultimately affects the cost of water purification. Although the FO process is expected to generally have a low surface fouling tendency compared to RO [22], membrane fouling/concentration polarization cannot be ignored in the FO process and needs to be mitigated/controlled to enhance process performance and achieve better water flux [23].

Various techniques in seawater desalination have been reported to mitigate and control FO membrane fouling/CP, and thus, enhance process performance. These include conventional and non-conventional pre-treatment systems, use of chemical dosing, membrane surface modifications, optimization of system operating conditions, and proper selection of the draw solution [23].

The use of ultrasound has been recently proposed as a pretreatment system or membrane cleaning technique for water and wastewater membrane treatment and desalination applications that could enhance membrane system performance. It should be noted that the use of ultrasound needs to be carefully selected because applying ultrasound at certain frequencies and arrangements can damage the membranes [24]. The use of ultrasound for membrane cleaning and flux enhancement has been recently considered for the FO osmosis process but it has received less attention and requires more investigation for process enhancement.

## 2. Forward Osmosis Systems

**Figure 1:**
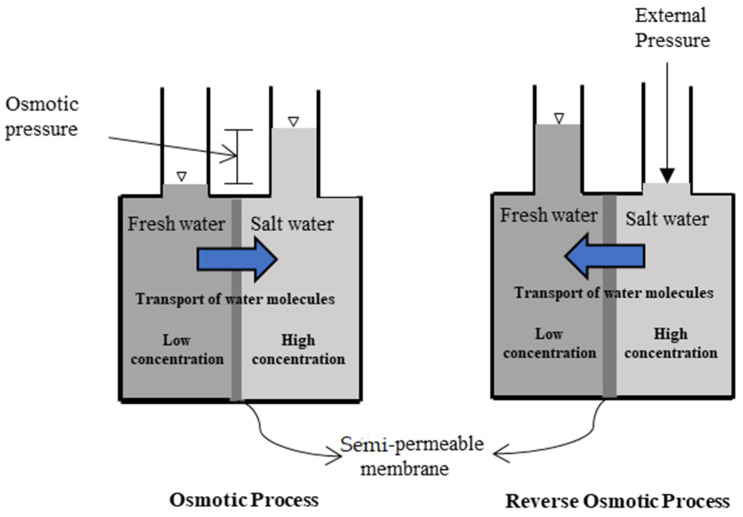
Schematic diagram of water flow in FO and RO processes.

**Figure 2:**
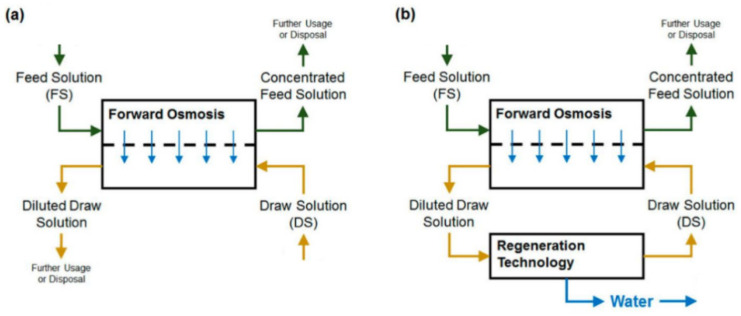
Schematic diagram of the FO process (**a**) without draw solution regeneration, and (**b**) with draw solution regeneration [17].

A forward osmosis (FO) membrane system is an evolving liquid membrane separation process, where the water molecules transfer across a semipermeable membrane utilizing the difference in the osmotic pressure (Δπ) between the two solutions located at each side of the membrane. The water movement occurs from a low concentration solution (i.e., low osmotic pressure), technically known as the diluted feed solution, towards a highly concentrated solution (i.e., high osmotic pressure), named the draw solution. The membrane permits only water molecules to be transferred, by diffusion, while the salts and all other contaminants are left behind; therefore, the concentration of salts in the feed solution increases while it decreases in the draw solution [25]. For the sake of comparison, the RO process requires external hydraulic pressure to be applied on the feed water side for process operation, which should be greater than the feed water osmotic pressure to force water flow in the reverse direction. However, the water molecules in the FO process move in an opposite direction to that of the RO process [26]. Figure 1 schematically shows the water flow in FO and RO processes. It must be noted that the water produced from the FO process is not pure water as it becomes part of the draw solution and thus requires an additional treatment process to extract the water from the draw solution to prepare it for use [27]. Such methods need to be carefully selected, as it is highly dependent on the type of the draw solution used and the associated re-concentration method cost [27]. Several methods have been reported in the literature for draw solution regeneration, including precipitation for draw solutions containing CuSO_4_ [7]; an MD process for 2-methylimidazole-based draw solution compounds [8,28]; UF for polyelectrolyte draw solutions (polyacrylic acid sodium salts) [9]; NF for solutions containing Na_2_SO_4_ [10], NaCl, MgCl_2_, Na_2_SO_4_, CaCl_2_, MgSO_4_, KCl, and C_6_H_12_O_6_ [11]; and RO for real seawater (Red Sea) [12], CaCl_2_, Ca(NO_2_)_3_, KBr, KCl, K_2_SO_4_, KHCO_3_, MgCl_2_, MgSO_4_, NaCl, NaHCO_3_, Na_2_SO_4_, NH_4_Cl, NH_4_HCO_3_, and (NH_4_)_2_SO_4_ by modeling the RO process using RO system design software [13]. Figure 2 illustrates the FO process without draw solution regeneration/recovery (a) and with draw solution regeneration/recovery (b).

**Figure 3:**
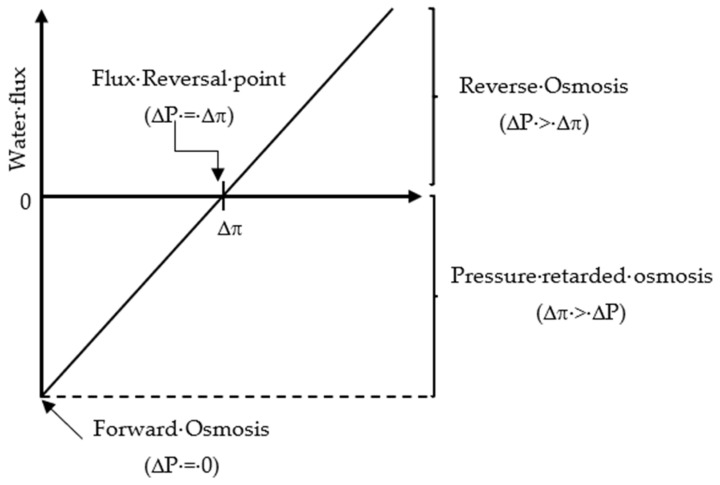
Relationship between the osmotic pressure processes (FO, PRO, and RO) in terms of flux, osmotic pressure difference, and applied pressure.

Based on the value of the applied pressure across the semipermeable membrane, the osmosis processes (OP) can be divided into three categories [29]: (1) FO when ΔP equals zero;(2) pressure-retarded osmosis (PRO) when ΔP is less than the system osmotic pressure difference (Δπ); (3) RO when ΔP is greater than Δπ. The relationship between the FO, PRO, and RO processes in terms of system flux, osmotic pressure difference, and applied pressure is shown in Figure 3.

**Figure 4:**
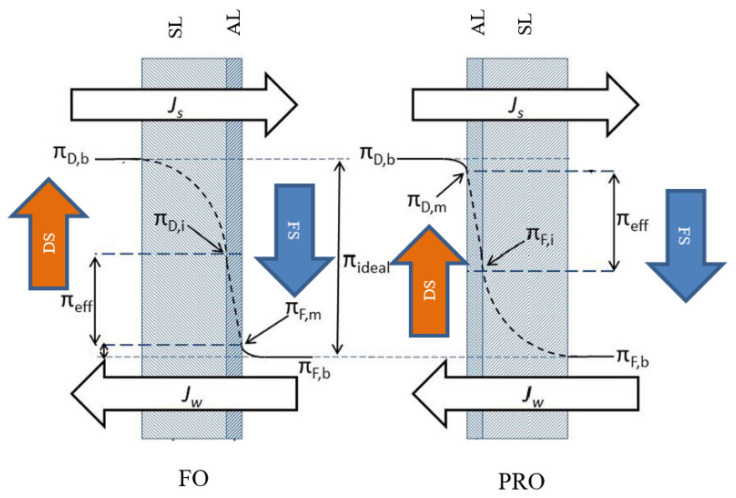
FO modes of operation [27] (Permission No. 5322071342556): FS, feed solution; DS, draw solution; AL, active layer; SL, support layer; *J_w_*, water flux; *J_s_*, solute flux; *π_D,b_*, bulk osmotic pressure of draw solution; *π_F,b_*, bulk osmotic pressure of feed solution; *π_D,i_*, osmotic pressure at the support layer adjacent to the active layer of draw solution side; *π_F,m_*, osmotic pressure at the active layer of feed solution side; *π_D,m_*, osmotic pressure close to the active layer of draw solution; π_F,I_, osmotic pressure at the support layer adjacent to the active layer of feed solution side; *π_eff_*, effective osmotic pressure.

Typically, two modes of operation can be configured in the FO process based on the orientation of the membrane layers. In the first mode, the membrane active layer faces the feed solution and the support layer faces the draw solution side; in the second mode, the feed solution faces the support layer and the draw solution faces the active layer [27]. Figure 4 illustrates these two FO modes of operation.

The FO process has many advantages compared to the alternatives. It operates with minimal or no hydraulic pressure requirements [14], which ultimately reduces it operating cost provided that the draw solution regeneration process is selected properly [22,30,31,32,33]. The process has relatively low fouling potential compared to RO since it does not require an external pressure force to be applied and fluid movement is mainly dictated by the osmotic pressure difference between the draw and feed solutions [14,17]. An FO system also shows a high water flux recovery rate after proper membrane cleaning [14,27], and can stand high salinity water (such as RO brine) without compromising the quality of the permeate [22]. Another RO feature is due to use of one of its alternative configuration (PRO), the process could generate power by utilizing the difference in osmotic pressure between seawater and freshwater [33]. The advantages, challenges and various applications of FO, as well as the challenges in selecting the proper draw solution for FO and effect on transport limitations in FO processes have been reviewed by Mohammadifakhr et al. [34].

### 2.1. FO Process Research Trend

**Figure 5:**
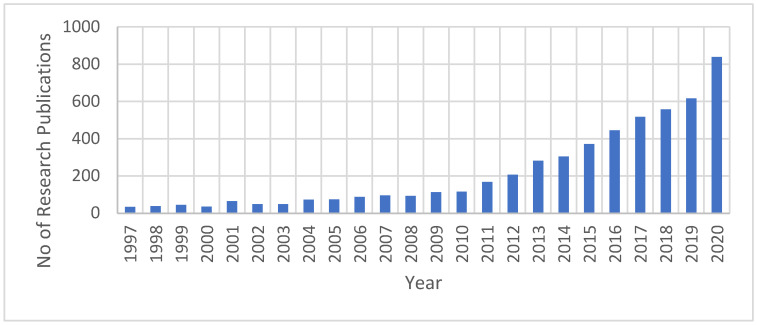
Growth in FO research publications as obtained from the Science Direct database.

The research efforts related to FO have been significantly increased, especially in the last ten years. Based on information obtained only from the Science Direct database when ‘’forward osmosis’’ was used as a keyword, there have been 5274 research publications related to the FO field since 1997 (Figure 5). From 2019 to the end of 2020, 1456 research publications were produced, representing around 28% of the overall publications since 1997. Thus, research efforts on FO technology have increased significantly in the past decade. This indicates the importance of both FO technology to the water industry and its characteristics that attract researchers for the investigation and development of process enhancements.

Suwaileh et al. [14] performed an analysis of the published research topics related to the FO process (from 1999 to 2020) and found that researchers focused on studies related to FO membrane fouling, energy consumption, draw solution types, draw solution recovery systems, membrane modifications/fabrications, techno-economy analysis, and mathematical modeling. The analysis showed that studying membrane fouling received the highest number of publications followed by draw solution types and membrane modifications/fabrications.

Researchers have conducted several studies on the fabrication of FO membranes by utilizing different types of novel materials, including polymers, modifying the membrane support layer structure by using different fabrication techniques, or modifying the characteristics of the membrane active layer that ultimately contributes to membrane performance [35].

Minimizing the effect of membrane fouling on membrane performance has been investigated by researchers, who have assessed operational parameter optimization, different types of cleaning techniques [14], fabrication of low fouling FO membranes from nanocomposite materials (zwitterion-silver) [36], and membranes with high hydrophilicity characteristics from incorporated hydrophilic nanomaterials [37]. Yan-jun et al. performed a thorough literature review on fouling and cleaning of membrane [38].

Many studies have been conducted by researchers to find and develop a draw solution that enhances the performance of the FO process. Many different types of draw solutions have been investigated, such as gaseous and volatile compounds, organic and inorganic compounds, functionalized nanoparticles (magnetic nanoparticles), and organic ionic salt [39].

### 2.2. Applications of FO in Water Desalination

The FO process has many applications, such as water desalination (brackish and saline water) [10,40,41,42], heavy metal removal [43,44,45], wastewater treatment and reclamation [46,47,48], power generation [49], the fertilizer industry, and food and beverage processing [50,51,52]. As already mentioned, the FO process has many advantages including minimal or no hydraulic pressure requirements [14] that ultimately reduces the operating cost provided that the draw solution regeneration process is selected properly [22,32].

Kravath and Davis in 1975 [41] were the first investigators to consider the possibility of using FO technology for desalinating seawater. They utilized cellulose acetate (CA) as the FO membrane. Seawater solution was used as a feed while the draw solution consisted of glucose (hypertonic). Results of the study proved the possibility of treating seawater with the FO process.

Zhao et al. [10] investigated the possibility of using a hybrid system combining the FO processes with nano filtration for desalinating brackish water. They tested two types of draw solutions (Na_2_SO_4_ and MgSO_4_) and found that the utilized systems had low NF membrane fouling and more than 90% water recovery was achieved.

McCutcheon et al. [42] investigated the applicability of using different concentrations (from 0.05 M to 2.0 M) of sodium chloride (NaCl) as a feed solution in the FO seawater desalination process by utilizing a FO polymeric membrane and ammonia-carbon dioxide as a draw solution. The authors found that the system was capable of achieving 95–99% salt rejection.

McCutcheon et al. [53] also investigated the use of different concentrations of NaCl as the feed solution in a FO seawater desalination process by utilizing a FO flat-sheet CTA membrane and ammonium bicarbonate (NH_4_HCO_3_) as the draw solution. Their results showed that a high salt rejection rate (about 95%) could be achieved.

Based on these studies, it can be said that developing forward osmosis membrane technology for water desalination has gained growing interest for what is considered a potentially energy-efficient process. However, the technology still faces serious limitations related to performance of the draw solution and membrane types, which are the main elements of the FO process. Another challenge involves the lack of an energy-efficient recoverable draw solution, which still consumes energy during the regeneration process.

### 2.3. Membrane Types and Designs

The old forms of FO membranes were manufactured from different types of materials, such as animal bladders, porcelain, and rubber [54]. After that, researchers utilized RO membranes for the FO process, where the use of asymmetric aromatic polyamide RO membranes was initially investigated by Loeb et al. [55].

Similar to RO membranes, FO membranes are composed of a thin dense layer, known as an active or rejection layer, and a porous support layer. However, the design approach is different because the support layer in an RO membrane needs to be thicker than the one in a FO membrane to maintain its structural integrity when hydraulic pressure is applied [29]. To create an efficient FO membrane, the active layer needs to be very thin with no defects in order to reduce flow resistance and increase membrane selectivity, which is highly dependent on the characteristics of the solute [29]. The support layer needs to be specially designed to provide maximum porosity, minimum tortuosity, with a minimum allowable thickness to ensure that the required mechanical strength is achieved [29].

FO membranes are generally constructed from asymmetric compounds. The top thin film layer, i.e., the rejection or active layer, has a thickness ranging from 100 to 200 nm. This rejection layer is connected to another layer at the bottom of the membrane, i.e., the support layer, having a thickness ranging from 100 to 200 μm. The support layer provides the required mechanical support for the membrane structure [56].

**Figure 6:**
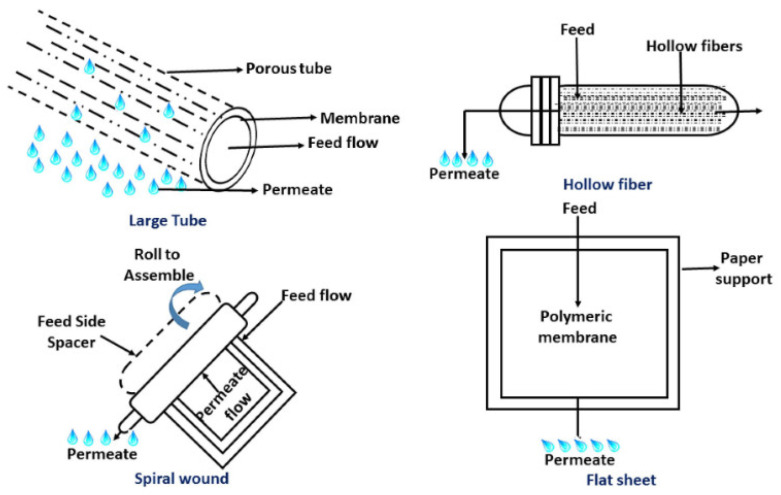
FO membrane configurations [58] (Permission No. 5322090221420).

Based on its geometry, FO membranes are available in different types, such as a flat sheet, plate or frame; tubular or hollow fibers; or spiral wound modules. Each type has its own advantages and disadvantages for the performance of FO processes [57,58]. Figure 6 shows the different configurations of FO membranes. FO membrane geometry plays an important role in selecting the proper application. For example, in wastewater treatment applications, where an extremely viscous or highly concentrated flow is required to be treated, flat sheet membranes are recommended. It has been reported that thin-film composite flat sheet FO membranes have higher performance in terms of flux and salts rejection in comparison to spiral wound and hollow fiber types when using sodium chloride as a draw solution [59]. For treating large seawater volumes, hollow fiber FO membranes are recommended [60], but they show low water flux and salt rejection [59].

The two main types of FO membranes commonly used in FO applications are cellulose acetate (CA)/cellulose triacetate (CTA) and thin film composites (TFC) [57].

#### 2.3.1. Cellulose Acetate (CA)/Cellulose Triacetate (CTA) Membranes

CA/CTA FO membranes are fabricated by utilizing the phase inversion method, which involves immersion of cast polymer dope into coagulants. Therefore, an asymmetric membrane structure is formed accordingly [61]. Hydration Technology Innovations (HTI) LLC produced the first commercial CTA FO membrane [54]. CA membranes have several advantages compared to other types, including good mechanical strength, worthy hydrophilicity properties, low tendency to fouling, chlorine tolerant, and ease for scaling up [32,35,62]. CTA membranes work properly in a pH range of 3 to 8 [63], though the manufacturer should be consulted for the best operating range. The available commercial CA/CTA membranes face several concerns, such as low water permeability, low solute rejection, limited chemical resistance, and weak resistance to biological foulants [14]. CTA FO membranes have been tested in a water desalination process utilizing the osmotic pressure generated from different draw solution types [35]. Table 1 provides applications for some CA/CTA FO membranes, as reported in the literature.

#### 2.3.2. Thin Film Composite (TFC) Membranes

TFC membranes were initially developed and produced by HTI, which also produced the first commercial CTA membrane, followed by Oasys water [64]. TFC asymmetric FO membranes are composed of thin support layer implanted with a mesh to provide adequate strength and support [35] and are fabricated according to the interfacial polymerization (IP) method. Compared to CTA membranes, TFC FO membranes demonstrated high water flux, high resistance to biodegradation, high salt rejection, etc. [77]. In addition, TFC membranes can function in a wider pH range (2 to 11) than CA/CTA. Moreover, the hydrophilicity characteristics of the membrane support layer associated with an interfacial polymerized TFC membrane can enhance water flux and reduce the internal concentration polarization (ICP) effect after decreasing the membrane structural parameter by utilizing different types of manufacturing materials and methods [29].

### 2.4. Draw Solution

The draw solution is considered one of the most critical parameters for FO performance. Therefore, selecting a proper draw solution type is mandatory to ensure an efficient treatment process [59,78]. A proper draw solution must have certain characteristics, such as a high osmotic pressure, low reversal flux, easily recovered, low viscosity, high solubility, high diffusion coefficient (low molecular weight), non-toxic, and be cost-effective [79,80]. Draw solution diffusivity and viscosity characteristics are crucial for FO system performance, since a highly diffused draw solution into the membrane support layer resulted in a reduction in the respective system internal CP and thus enhances the membrane performance [80]. Draw solution viscosity needs to be low to facilitate pumping and enhance water flux [27]. Table 2 displays the main characteristics of the draw solution and their effects on membrane water flux [14,80].

The most tested draw solutions for FO processes can be divided into two categories, namely, inorganic and organic compounds. However, other special types of draw solutions have also been investigated, such as ionic liquid and functionalized nanoparticles compounds [14].

#### 2.4.1. Inorganic Compounds

Generally, inorganic compounds including monovalent and multivalent metal ion compounds are considered the most commonly used draw solutions for FO processes [63]. Monovalent compounds have several characteristics that facilitate their use in the FO process, such as low cost, availability in large quantities, high flux due to their high osmotic pressure value, high solubility, low viscosity, and high diffusion coefficients that minimize the CP effect. However, the small molecular size associated with monovalent ions leads to an increase in reverse solute flux [14]. Several monovalent compounds have been studied and tested by researchers, such as NaCl, KCl, KBr, KHCO_3_, NaHCO_3_, KNO_3_, NaNO_3_, and NH_4_Cl [13,81,82,83]. It should be noted that reverse solute flux is an important aspect and needs to be controlled. She et al. [84] investigated the effect of reverse solute flux on membrane organic fouling during PRO operation. They found that a draw solution with a high concentration of divalent ions such as magnesium and calcium leads to major alginate biofouling in the presence of reverse solute flux. They found also that higher draw solution concentrations increased reverse solute flux.

Multivalent compounds, such as MgCl_2_, CaCl_2_, MgSO_4_, Na_2_SO_4_, and CuSO_4_, provide some advantages over monovalent compounds. They provide high osmotic pressure and low reverse solute flux [7,13,85]. An NaCl solution is commonly used in research experiments due to its high solubility and the ability to re-concentrate by utilizing the RO process with no scaling risk [54].

Achilli et al. [13] investigated FO membrane performance in terms of water flux and reverse solute flux for 14 types of inorganic draw solution compounds in desalination applications. The tested compounds were CaCl_2_, Ca(NO_2_)_3_, KBr, KCl, K_2_SO_4_, KHCO_3_, MgCl_2_, MgSO_4_, NaCl, NaHCO_3_, Na_2_SO_4_, NH_4_Cl, NH_4_HCO_3_, and (NH_4_)_2_SO_4_. They found that draw solutions having large ions, namely, NaHCO_3_, Na_2_SO_4_, KHCO_3_, K_2_SO_4_, (NH_4_)_2_SO_4_, and MgSO_4_, showed less reverse solute behavior.

Ansari et al. [86] investigated the use of seawater as a draw solution in the FO process for the recovery of calcium sulfate from a digested sludge centrate without the need for chemical additions and draw solution reconcentration. They found that that the pre-concentration of the centrate by three-times allowed the FO process to recover around 92% of phosphate by precipitation and the formed membrane fouling was reversible. Alnaizy et al. [7] tested the use of CuSO_4_ as a draw solution in the FO process for desalinating brackish and sweaters. They found that the maximum osmotic pressure generated by CuSO_4_ (2.99 MPa) was unable to be used for seawater desalination. However, CuSO_4_ was found to be sufficient for brackish water desalination applications. The effect of the draw solution molecular weight on FO membrane water flux has been investigated by Yasukawa et al. [87]. Two types of DS with different molecular weights were investigated, namely, NaCl and polyethylene glycol (PEG). They found that increasing the draw solution molecular weight from NaCl to PEG led to a significant decrease in membrane water flux due to ICP attributed to the low diffusivity associated with a large molecular weight draw solution solute.

Holloway [88] investigated the effect of using a mixed draw solution containing NaCl and MgCl_2_/MgSO_4_ on FO process performance, i.e., for minimizing reverse solute flux and high water flux. They found that divalent ions resulted in lower flux compared to monovalent ions at an equal osmotic pressure. Moreover, the mixed draw solution was found to be more effective for reducing reverse solute flux. Alejo et al. [89] reported that using multivalent compounds such MgCl_2_ and CaCl_2_ significantly minimized reverse solute flux, which was attributed to the large hydration radius and high electrostatic repulsion of the solutes. The effect of mixing two draw solutions on FO membrane performance was investigated by Nguyen et al. [90], where a small amount of Al_2_(SO_4_)_3_ was added to MgCl_2_ and mixed at a pH of 6.5. An FO CTA nonwoven membrane was utilized, and brackish water (5000 mg/L NaCl) and seawater (35,000 mg/L NaCl) were used as the feed solutions. The results showed that the mixed solution enhanced the system performance by reducing the reverse solute flux effect. Achilli et al. [13] reported that when the feed solution or draw solution contains calcium or magnesium ions, a scaling problem is expected to arise on the membrane surface. However, utilizing Al_2_(SO_4_)_3_ in the draw solution will minimize this effect.

#### 2.4.2. Organic Compounds

Unlike the inorganic ionic compounds, organic compounds generate low water flux under the same operating conditions. However, these compounds reveal high salts rejection during the reverse osmosis regeneration process [29]. Similar to the inorganic compounds, high molecular weight organic compounds are associated with low reverse solute flux [27], but they are expensive compared to inorganic compounds [13]. Phuntsho et al. [83] tested nine different fertilizers as draw solutions for desalinating brackish water. The authors found that KCl, NaNO_3_, and KNO_3_ were more efficient draw solutions among the other compounds in terms of water flux, and that each 1 kg of fertilizer was able to desalinate around 11 to 29 L of seawater.

### 2.5. Process Performance

Generally, the performance of the FO process is measured by the output capacity, final draw solution concentration, and the recovery rate of the feed solution. These indicators are mainly dictated by several parameters and the system operating conditions [91].

As discussed earlier, several factors affect the FO process performance, which are mainly dictated by the FO membrane characteristics and the system operating conditions. The ideal FO membrane should be capable to provide a high level of water flux production, high solute rejection at both sides of the membrane, minimal concentration polarization, strong membrane mechanical structure, low fouling behavior, and high chemical resistance [92]. The system operational parameters that play an important role in membrane performance include flow velocity, feed and draw solution concentration, flow configuration, temperature, and pH [23]. The influence of cross flow velocity and flow configuration on the FO membrane process are reviewed in the forthcoming sections.

#### 2.5.1. Cross-Flow Velocity

The cross-flow velocity can be defined as the system linear flow velocity that flows tangentially to the membrane surface area, and it can be mathematically calculated by dividing system flowrate by the channel cross-sectional area [93]. The cross-flow velocity related to a FO system plays an important role in process performance. Increasing the flow velocity enhances the mass transfer coefficient that ultimately minimizes the external concentration polarization (ECP) effect, and thus, increases the water flux accordingly [94]. Low cross-flow velocity reduces reverse solute transfer from the DS side towards the FS. However, low cross-flow velocity increases the chance for the ECP effect and promotes membrane fouling [95]. A higher feed solution cross-flow velocity and a lower draw solution cross-flow velocity maximizes the dilution of the draw solution and minimizes the CP and fouling effects on the feed solution side. However, a small difference between the cross-flow velocities of the feed and draw solutions has an insignificant effect on ECP and mass transfer rates [96]. The draw solution cross-flow velocity also needs to be sufficient to prevent the negative effect of draw solution dilution [21]. It has been reported that a high cross-flow velocity ratio for the feed solution and the draw solution leads to additional stresses on the membrane surface, and thus, increases the potential for damaging the membrane [97].

Devia et al. [95] investigated the effect of cross-flow velocity on CTA FO membrane water flux using MgCl_2_ as a draw solution for treating secondary effluent. The study revealed that a high cross-flow velocity leads to higher water flux. Meanwhile, Phuntsho et al. [82] evaluated the effect of some of the major parameters that affect the FO process, such as membrane properties, draw and feed solution characteristics, and the operating conditions. They found that higher water flux could be obtained by increasing the cross-flow velocity. Tran et al. [98] considered pressure retarded osmosis (PRO) by using a semipermeable membrane separates two solutions: the draw solution, with higher salinity, and the feed solution, with lower salinity. They found that the water flux behavior at high hydraulic pressure difference is shown to be nonlinear, departing from the theoretically predicted water flux, which is based on a linear model. Lotfi et al. [99] studied the effect of several operating factors, such as cross-flow velocity, pH of the feed solution, and system pressure, on system behavior using synthetic seawater as the draw solution (35,000 mg/L) and synthetic wastewater as the feed solution. CTA flat sheet and polyamide TFC FO membranes were tested. They observed a fluctuating (sinusoidal) water flux pattern, which was attributed to the formation of a fouling layer on the membrane surface. They also observed that a high cross-flow velocity enhanced removal of the fouling layer from the membrane surface at a certain layer thickness.

#### 2.5.2. Flow Configuration

Two flow configurations are typically used in FO systems, namely, co-current (the feed solution and draw solution enter the system on the same side) and counter-current (the feed solution enters the system on one side and the draw solution enters at the other side) configurations [100]. Operating a hollow fiber FO system under a counter-current arrangement maintains a constant osmotic pressure difference between the feed solution and the draw solution [101].

Deshmukh et al. [102] investigated the effect of TFC membrane characteristics, such as water and solute permeability, structural parameter, and membrane flow arrangements, such as co-current and counter-current, on overall membrane performance by utilizing module scale modeling. Higher water recovery was achieved by utilizing counter-current flow as compared to a co-current arrangement. Kim et al. [103] investigated the effect of system operating conditions on FO TFC spiral wound membrane performance. The study revealed that a co-current configuration promoted membrane fouling when the system was operated at high flow rates. Mondal et al. [104] developed an analytical solution for sizing the FO membrane system (membrane area) after considering co-current and counter-current flow arrangements based on the log-mean driving force (concentration difference) method. Similarly, Banchik et al. [105] investigated and developed an analytical model for FO membrane sizing by considering counter-current and co-current flow arrangements and the CP effect (internal and external) for FO and assisted FO systems used in fertigation, pharmaceutical, and resource extraction applications.

Shim and Kim [106] developed a numerical model for the effect of FO system operating parameters, such as water flux and recovery, concentration distribution over the membrane area, and co-current and counter-current flow configurations on membrane performance. The results showed that counter-current flow produced around 10% more water flux than the co-current arrangement.

Phuntsho et al. [83] investigated the effect of flow configuration and crossflow velocity on FO (CTA) membrane performance using brackish water and deionized water as feed solutions and KCl as the draw solution. They observed no significant effect of the flow configuration on water flux, and this was attributed to the small size of the testing cell (L = 7.7 cm, W = 2.6 cm, and H = 0.3 cm).

## 3. FO System Challenges

When considering the FO process in seawater desalination applications, the main challenges involve fouling of FO membranes and CP problems [14].

### 3.1. Concentration Polarization

**Figure 7:**
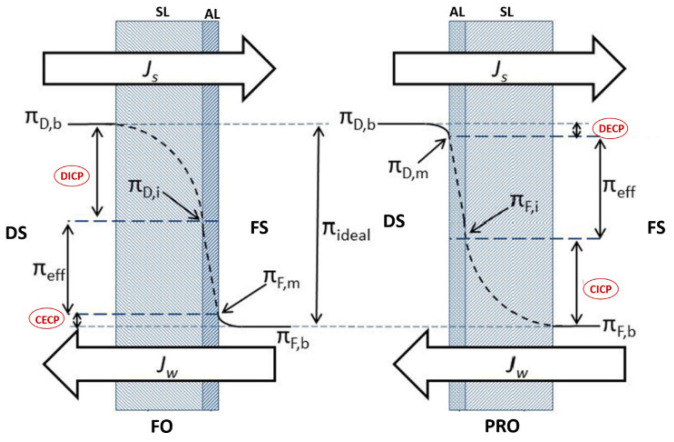
Internal and external concentration polarization types [27].

Concentration polarization (CP) phenomena is a process that occurs in all crossflow membrane desalination systems such as RO and FO systems. It negatively impacts the system operating performance by reducing water flux due to a concentration gradient between the system solutions (feed solution and draw solution) and the FO membrane rejection layer [107]. Based on its occurrence, CP can be categorized into internal concentration polarization (ICP) and external concentration polarization (ECP) [27]. ICP occurs within the membrane support porous layer (SL), whereas ECP occurs at the surface of the membrane active layer (AL) [22]. ECP and ICP can be further classified, based on the active layer orientation, into dilutive or concentrative concentration polarization. Dilutive external concentration polarization (DECP) and concentrative internal concentration polarization (CICP) occurs when the membrane active layer faces the draw solution, whereas concentrative external concentration polarization (CECP) and dilutive internal concentration polarization (DICP) occurs when the active layer faces the feed solution [82]. Figure 7 illustrates ICP and ECP in the case of FO and PRO.

ICP is considered one of the most critical concerns related to FO membrane performance in terms of permeate flux reduction. The effect of ICP cannot be easily eliminated by manipulating the system operating conditions [108]. Zhao and Zou [109] have reported that ICP in the membrane support layer when the feed solution faces the membrane active layer is mainly dictated by the draw solution characteristics. They reported that draw solutions with low diffusivity, high viscosity, and a large molecular size cause severe ICP that adversely impacts membrane water flux. Table 3 summarizes the types of CP based on orientation of the active layer.

### 3.2. Scaling and Fouling

**Figure 8:**
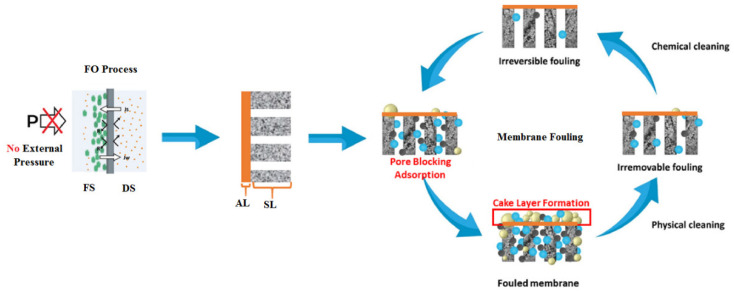
External and internal FO membrane fouling types [58]. FS, feed solution; DS, draw solution; AL, active layer; SL, support layer.

Membrane fouling occurs due to the presence of several contaminants that typically exist in the seawater feed, such as organic matter, inorganic and scaling substances, colloidal and suspended particles, and biological foulants [14]. Based on the occurrence, FO membrane fouling can be categorized into external and internal fouling. External fouling, sometimes called surface fouling, occurs at the external surface of the membrane active layer facing a seawater feed solution when a cake layer forms on the membrane surface. Internal fouling occurs when undesired deposits accumulate inside the membrane pores and may result in membrane clogging, as is the case when the active layer of the membrane faces the draw solution [23]. Figure 8 illustrates the two types of FO membrane fouling.

It is known that the FO membrane configuration plays a vital role in its water flux performance as it significantly affects the formation of fouling and CP layers. It has been reported that membranes having an active layer facing the feed solution normally have a lower fouling/CP potential, and thus, provide higher permeate water flux compared to a membrane where the active layer faces the draw solution [110].

Organic fouling of FO membranes is influenced by the interaction between the foulant substances in the feed solution and the membrane surface [111]. Sodium alginate is commonly used to simulate organic fouling [112].

A biofilm formation layer on FO membranes is more complicated compared to those found in RO systems [113,114]. Highly concentrated draw solutions that contain divalent cations promote the reverse salt flux problem, which facilitates the interaction between solute particles and the biofilm layer that ultimately causes flux reduction [115].

She et al. [113] reported that the availability of a high concentration of scale-causing ions, such as sulfate, calcium, or magnesium, in the draw solution promotes membrane scaling and organic fouling when these ions diffuse inversely into the feed solution. This process ultimately reduces the feed solution osmotic pressure, and thus, reduces membrane water flux. Zou et al. [114] investigated the effect of the presence of microalgae in feed water on FO membrane performance when utilizing NaCl and MgCl_2_ as draw solutions. They found that severe microalgae fouling occurred in the presence of divalent cations such as magnesium.

Liu et al. [116] studied the effect of the combination of organic foulants with scalants on FO membrane flux. Sodium alginate and calcium sulfate were tested on a CA FO membrane and the results revealed that alginate facilitates an increase in the crystal sizes of the scalants to reduce water flux.

Nguyen et al. [117] investigated the effect of the availability of different fouling substances in the feed solution on FO membrane flux. The authors considered the effects of single and combined foulants on flux. CTA FO polyester woven mesh and PA-TFC membranes were used. Sodium alginate (200 mg/L) was used as an organic foulant and CaCl_2_ (20 mM) and Na_2_SO_4_ (20 mM) were used to represent gypsum scaling. The results showed that in a single foulant test (sodium alginate), both membranes (CTA and TFC) had higher flux values of 15.9 and 20.5 L/m^2^·h, respectively. However, the effect of combined foulants (sodium alginate and gypsum) was found to be more severe, where the flux for each membrane (CTA and TFC) was 5.4 and 8.3 L/m^2^·h, respectively.

Liu and Mi [118] utilized sodium alginate and gypsum to study the synergistic effect of the presence of organic and inorganic foulants on CA FO membrane performance. The draw solution consisted of 4 M NaCl. They found that the presence of sodium alginate in the feed solution accelerated gypsum scaling on the membrane surface, ultimately reducing water flux. They also reported that the size of the gypsum scaling crystals was also influenced by the presence of sodium alginate by providing a nuclei for the gypsum crystallization process.

### 3.3. Reverse Solute Flux

Reverse solute flux (RSF) is considered also has a profound effect FO membrane process performance [119]. An FO membrane and FO operation should only allow the transfer of the solvent (i.e., water in case of desalination) from the feed solution side to the draw solution while preventing the transfer of other solutes in any direction [113]. Several models have been reported in the literature to explain RSF. Generally, the basic RSF ($J_{s})$ model follows Fick’s law, as shown in Equation (1) [27]:

(1) $$J_{s}=B\Delta C$$

where *B* is the membrane solute permeability coefficient, and ΔC is the concentration difference across the membrane active layer.

Phillip et al. [120] utilized a CTA FO membrane and NaCl as a draw solution to develop an RSF model for the FO process without taking into account the effect of ECP at both sides of the membrane as follows:

(2) $$J_{s}=\frac{J_{w}\;C_{D,bulk}}{1-\left(1+\frac{J_{w}}{B^{\prime }}\right)\exp\left(\frac{J_{w\;}S}{D}\right)}$$

where $C_{D,bulk}$ is the draw solution bulk concentration, *S* is the FO membrane structural parameter, *D* is the effective diffusion coefficient, and *B’* is the membrane active layer permeability layer.

Several issues are associated with RSF in the FO process, including the promotion membrane fouling, loss of draw solution concentration (additional cost), and flux decline due to a reduction in osmotic pressure difference [32]. The reverse solute problem depends on several factors, some of which are related to the membrane characteristics, such as membrane porosity, thickness and tortuosity [107], and others related to the solute solution properties, such as ionic charge, viscosity, ionic radius, and diffusivity [107]. The draw solution ionic charge plays an important role in the RSF problem. After considering a surface negative charge for CTA and TFC membranes, it was demonstrated that positively charged ions had a higher tendency to penetrate through the membrane layer than negatively charged ions [121]. It is worth mentioning that diffusion of solute particles can occur in both directions across the semi-permeable FO membrane. However, the term RSF is related to solute permeation from the draw solution towards the feed solution [42]. During the FO process, using a draw solution with multivalent ions minimizes the RSF effect due to the large size of the ions and their low diffusion properties [70]. However, such characteristics promotes the ICP effect within the membrane SL [119]. Lay et al. [122] have reported that RSF had a significant impact on membrane performance that ultimately affected water flux.

## 4. Process Performance Enhancement

Various techniques in seawater desalination have been proposed to mitigate and control FO membrane fouling/CP, and thus, enhance process performance. These include conventional and non-conventional pre-treatment systems, chemical dosing, membrane surface modifications, optimization of system operating conditions, and proper draw solution selection [23]. Pretreatment of feed seawater ahead of the FO membrane would minimize the impact of fouling but may increase the overall footprint of the system, adding cost by increasing the overall power demand [14]. Volpin et al. [123] reported that a small increase in FO process flux could significantly enhance the investment rate of return. Since membrane fouling is significantly impacted by seawater characteristics, it is necessary to thoroughly investigate them in order to identify the most problematic parameters for membrane fouling so that the most effective mitigation technique is adopted.

Several studies have been conducted on FO membrane performance enhancement. Zhao and Zou [124] have investigated the adverse effect of high feed brackish water temperature on membrane performance and scale/fouling formation mechanisms for using real brackish water samples. They found that a high feed water temperature provided high initial flux and water recovery; however, higher temperatures promoted membrane scaling. Zhang et al. [125] have studied the effect of air cavitation on FO membrane bioreactor performance for wastewater treatment and achieved excellent system flux and fouling reversibility.

It has been reported that physical cleaning methods were not efficient foe controlling biofouling [126]. As such, chemical cleaning with sodium hypochlorite has been tested by Yoon et al. [126] and showed good results compared to physical cleaning. Lotfi et al. [99] studied the effect of cross-flow velocity on FO membrane water flux using a synthetic wastewater solution as the feed solution and seawater as the draw solution. They found that increasing the cross-flow velocity and applying a simple hydraulic washing regime and doubling the feed flow rate resulted in a significant positive impact on membrane flux recovery.

Xiao et al. [127] investigated the effect of different factors on FO membrane performance for FO and PRO modes of operation. They found that the temperature of the feed and draw solutions played a significant role in membrane performance, where increasing the draw solution temperature resulted in a decrease in water flux, and the PRO mode showed more fouling potential than the FO mode. Akther et al. [128] studied the effect of feed solution (seawater) and draw solution (NaCl) flow rates, and draw solution concentration and temperature on TFC FO membrane performance (co-current with the membrane active layer facing the draw solution). Enhanced water flux was achieved by increasing the flow rates for both the feed and draw solutions and increasing the draw solution concentration. The authors also reported that an increase in draw solution temperature had a slight enhancing effect on membrane flux.

Enhanced desalination performance of FO membranes based on reduced graphene oxide (GO) laminates coated with hydrophilic polydopamine has been considered by Yang et al. [129]. The authors applied chemical reduction of GO laminates and a hydrophilic adhesive polydopamine (pDA). According to the authors, reduced GO (rGO) laminates maintained the same nanochannel size (3.45 Å) compared to pristine GO laminates, which consequently increased its selectivity for hydrated ions. They also found that adding a pDA coating onto the rGO laminates improved its hydrophilicity, which consequently resulted in an increase in the rate of water absorption. Due to these effects, pDA-coated rGO (pDA-rGO) membranes achieved a water flux of 36.6 L/m^2^·h, with a reverse solute flux of 0.042 mol/m^2^·h and a high salt rejection rate of 92.0% during FO.

Hsu et al. [130] also developed a new approach to efficiently desalinate water via an FO process using a binary ion liquid/hydrogel system. Their study demonstrated that the hybrid ion liquid/hydrogel draw solution system synergistically leveraged the thermo-responsive properties of both the ionic liquid (IL) and hydrogel to improve overall FO performance. They found that hydrogels can be used in a continuous and readily recyclable process to recover water without heating the entire draw solute/water mixture. This would open the door to use low-grade/waste heat or solar energy to regenerate draw agents and potentially reduce energy consumption by the FO process considerably.

Enhancing the desalination performance of a FO membrane through the incorporation of green nanocrystalline cellulose (NCC) and halloysite dual nanofillers has also been investigated [131]. The enhancement of both the water flux and antifouling properties of the membrane was achieved without compromising salt rejection when a low amount of NCC (<0.1 wt/v%) was incorporated into the thin polyamide layer and demonstrated the potential of dual nanofiller thin film nanocomposites for effective forward osmosis desalination applications.

## 5. Ultrasound Technique in FO Process

Based on the previous discussion, it can be concluded that membrane fouling and CP decrease membrane productivity. Thus, in order to increase the membrane lifetime, it is necessary to find appropriate solutions for removing fouling and mitigation measures that maximize membrane productivity for a given capital and operational cost. Ultrasound technology has been recently proposed as a pretreatment system or membrane cleaning technique for water and wastewater membrane treatment and desalination applications that could enhance system performance. However, the use of ultrasound for membrane cleaning and flux enhancement for FO is still in the early stage and requires more investigation before it can be utilized for process enhancement.

Ultrasound, sometimes called ultrasonic wave, is an acoustic (sound) wave that is traveling at a frequency greater than 20 kHz [132]. Ultrasonic waves have a special ability to produce high mechanical power in a physical medium such as liquid, gas, or solid through multiple rounds of minor mechanical movement [24]. Sound wave propagation in a medium starts with a group of alternating compression and expansion (rarefaction) wave cycles. During the compression cycle, the pressure increases, and the distance between fluid molecules decreases, whereas the distance increases during the expansion cycles [133]. The cycles of compression and rarefaction creates acoustic pressure that is a function of wave amplitude, time, and wave frequency. Table 4 displays the different categories of ultrasound waves, their frequency ranges and associated applications [134]. Ultrasound waves with a frequency range of 20–500 kHz are commonly used in cleaning and industrial applications [24].

**Figure 9:**
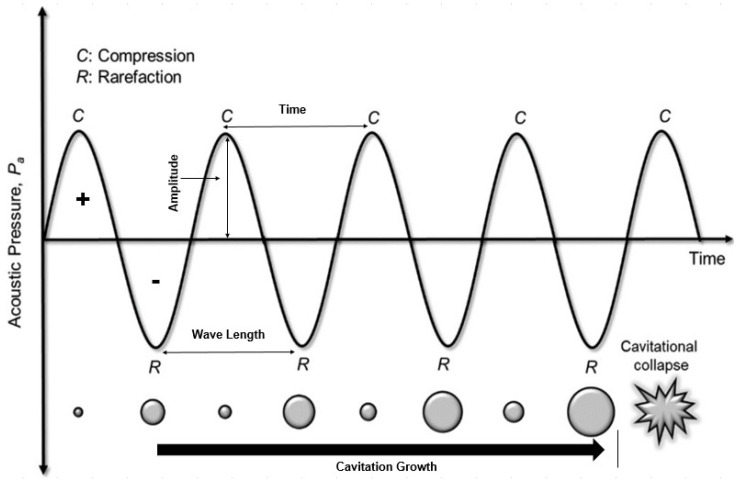
Ultrasound cavitation stages: acoustic cavitation, cavitation growth, and collapse [24] (Permission No. 5322081328929).

During the compression and rarefaction cycles, the molecules in the medium are exposed to a positive (compression) and negative (rarefaction) acoustic pressure. Positive pressure pushes the molecules closer whereas the negative pressure makes them expand, ultimately creating a phenomenon called bubble cavitation. Some of the created bubbles, supported by cyclic compression and rarefaction, combine to produce a new and larger bubbles in a process called bubbling growth. Bubbling growth keeps growing until it reaches a certain size and then collapses. Bubbling collapse produces extreme pressure and temperature, up to 1000 atm to 5000 K, respectively [134]. Figure 9 illustrates the ultrasound cavitation stages: acoustic cavitation, cavitation growth, and collapse.

Ultrasonic waves provide important mechanisms that can be used to clean fouled membranes, which will ultimately enhance membrane flux performance. These mechanisms include acoustic streaming (no cavitational collapse), microstreaming (shear effect), micro-streaming (wave superimposition), microjets (differential pressure), and shock waves [135]. Typically, two types of ultrasound equipment, namely ultrasonic probes and ultrasonic baths, are used for generating acoustic cavitation [136]. The effects of several operational parameters on the performance of an ultrasonic system have been addressed; these include system frequency (low frequency provides more cavitational collapse), ultrasonic intensity, external pressure, liquid temperature [24], and the operation mode (intermittent/continuous) [137]. Based on the advantages of the ultrasound technique, researchers have investigated its use for membrane cleaning/fouling controls and process performance enhancement (increasing flux) during certain membrane separation processes such as microfiltration (MF) and ultrafiltration (UF) [24]. However, studies related to use of ultrasound in FO systems, especially for seawater applications, are very limited and need to be expanded for better process performance.

Ultrasound-assisted FO systems have received limited attention in the literature. Heikkinen et al. [137] have studied the use of ultrasound for mitigating CP in a TFC FO membrane. Tannin (wood bark) solution was used as the feed solution (facing the active layer) and sodium sulfate as the draw solution (facing the support layer). The authors considered the effect of different ultrasound frequencies (20, 573, and 1136 kHz) and operating modes (continuous or intermittent) on process performance and found that when the ultrasound was applied from the feed side, flux increased by 1.2 times at 50 W and by 2.2 times at 100 W during a continuous operation mode. However, during an intermittent mode of 1 min on and 1 min off, they found that the flux increased by 2.1 times at 100 W and by 1.7 times when the intermittent operation involved 1 min on and 5 min off at 100 W. Moreover, the lowest used frequency of 20 kHz was found to be the most efficient.

The effect of using ultrasound during FO for fruit juice concentration was studied by Chanukya and Rastogi [138] using a CTA FO membrane and a 30 kHz ultrasonic path. Simulated fruit solutions (sweet lime and rose) were used as a feed whereas the draw solution consisted of 6 M NaCl arranged in a co-current flow mode. The authors found that when the process was assisted by ultrasound, the initial flux increased by 20% for the rose solution and about 30% for the sweet lime solution.

Nguyen et al. [139] studied the effect of using an ultrasound-assisted (40 kHz) FO system for sludge thickening process enhancement and membrane fouling reduction. An FO CTA flat sheet (rolled as a tube) membrane was used. Activated sludge was taken from a secondary clarifier along with some chemical additions to make synthetic sludge to be used as the feed solution. A NaCl solution of 36,000 mg/L was used as the draw solution and kept constant for the entire process. The authors found that ultrasound was able to achieve sludge concentration from 3 g/L to 20.4 g/L in 22 hr, whereas the time required to reach the same final concentration without the use of ultrasound was about 26 h.

Choi et al. [140] investigated the effect of utilizing ultrasonic waves (ultrasonic probe-72 kHz) for mitigating calcium sulfate scaling and colloidal (silica) fouling, individually, in a FO membrane system. Synthetic feed and draw solutions were prepared at concentrations of 0.5 M NaCl and 4 M NaCl, respectively. The authors considered the effect of applying an external hydraulic pressure (up to 5 bar) on the feed side and found that the initial membrane flux increased by about 25% (in the CaSO_4_ scaling test) when the ultrasound was applied to the FO mode, and about 166% for pressure when using the ultrasound combined mode. During the colloidal test, flux declined by 21% during the ultrasound-FO mode and about 19% in pressure for the ultrasound combined mode. They also reported that calcium sulfate scaling was reversible whereas colloidal fouling was more difficult to clean and caused a severe flux decline. It is worth mentioning that the effect of the combined mixture of CaSO_4_ and silica was not investigated.

Choi et al. [141] studied the effect of ultrasound on minimizing ICP during the FO membrane process using aCTA membrane and a counter-current flow configuration. A sodium chloride solution of 0.5 M was used as the system feed, and a 1–4 M NaCl solution was used as the draw solution. The temperature of both the feed and draw solutions was kept constant at 20 °C. The authors found that using ultrasound could significantly reduce the ICP effect and better membrane flux enhancement was obtained (about 81%) at a low ultrasound frequency of 25 kHz. No scaling or fouling materials were tested in this study to represent typical seawater characteristics.

The impact of ultraviolet/persulfate (UV/PS) oxidation pre-treatment for the mitigation of organic fouling during the FO process for anaerobically treated dairy effluent (ATDE) was investigated using a multi-cycle filtration method [142]. The authors compared the UV/PS performance with control pre-treatments such as stand-alone ultraviolet (UV) irradiation and potassium persulfate (PS) oxidation. Size exclusion chromatography confirmed that flux reduction over successive filtration cycles was due mainly to humic substances and building blocks, i.e., sub-units of humic substances in the feedwater. Although all investigated pre-treatment options mitigated membrane fouling, UV/PS achieved a greater enhancement in flux and decreases in both reversible and irreversible foulant deposition than the stand-alone UV and PS pre-treatments.

Qasim et al. [143] investigated the effect of using inorganic draw solutions (MgSO_4_ and CuSO_4_) and an ultrasound path on FO process performance with a CTA FO flat sheet membrane and a co-current flow arrangement. The process was tested by utilizing simulated brackish water and seawater as a feed solution. This was obtained by dissolving sodium chloride in deionized water (DI) to obtain a final feed solution of 5000 mg/L to represent brackish water and 40,000 mg/L to represent seawater. The testing was conducted at a fixed flow rate, constant feed, a draw solution temperature of 25 ± 1 °C, continuous ultrasound frequency of 40 kHz, and a continuous mode facing the membrane support layer. The results revealed that using either MgSO_4_ or CuSO_4_ as a draw solution at a certain concentration when assisted by ultrasound effectively minimized the ICP effect, and water flux was enhanced by 36.4% and 43.9%, respectively.

**Figure 10:**
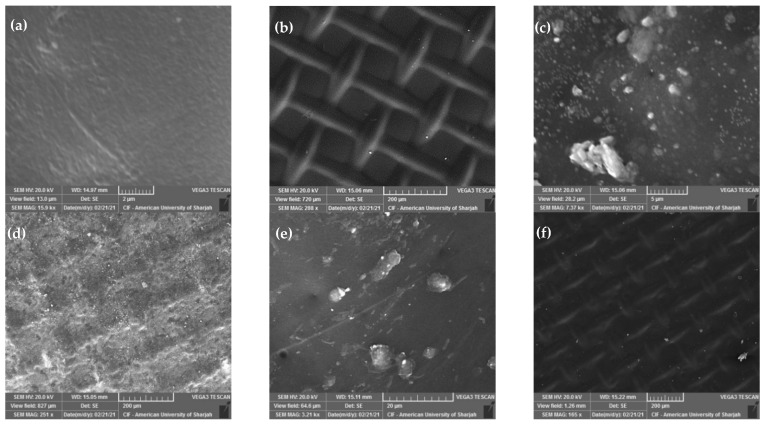
The effect of using ultrasound on water flux through a forward osmosis membrane during desalination of synthetic seawater. SEM images of the original membrane (**a**) active layer and (**b**) support layer; the fouled membrane after the use of a low CFV with the ultrasound facing the support layer (Case 0.25-CC-SL with NaCl draw solution) (**c**) active layer and (**d**) support layer; and the fouled membrane after the use of a high CFV with the ultrasound facing the active layer (Case 1.0-CC-AL with MgCl_2_ draw solution) (**e**) active layer and (**f**) support layer [144].

The authors of this review have recently investigated the effect of using ultrasound on water flux through a forward osmosis membrane when applied to seawater desalination [144]. A synthetically prepared solution simulating seawater with scaling substances and organic foulants was used. Parameters considered include membrane crossflow velocity, flow configuration (co-current versus counter-current), direction of ultrasound waves relative to the membrane side (active layer versus support layer), and type of draw solution (NaCl versus MgCl_2_). Examples of SEM images of the fouled membranes obtained at low and high cross flow velocity (CFV) are shown in Figure 10. The figure demonstrates an increase in membrane fouling when the ultrasound was facing the support layer and at low cross flow velocity (Figure 10c,d) compared to when the ultrasound was facing the active layer with a high cross flow velocity (Figure 10e,f). Details of the results and findings are described elsewhere [144].

## 6. Future Directions

Based on the limited work reported in the literature, it appears that coupling ultrasound with FO would enhance water flux through the membrane under certain conditions. However, there is a need for further research to address several questions related to utilization of a coupled ultrasound/FO system for seawater desalination. As of today, no work has been conducted to assess the use of ultrasound on FO membrane flux and concentration polarization with real seawater (or even for synthetic seawater that contains scalant and fouling materials). Therefore, studies are needed in this regard to assess the effect of operating parameters on the performance of the FO system. Parameters that could be investigated include crossflow velocity of the feed and draw solution, flow configuration, ultrasound source location, ultrasound operation mode (intermittent/continuous), and ultrasound frequency. Verification of flux enhancement with the use of ultrasound should be coupled with a detailed investigation of its effect on membrane resistance through microscopic observations of deposited foulants on the membrane. Knowledge about the surface and cross-section morphologies of the membrane as well as the amount and compositions of the deposited foulants should provide a better understanding of the role of ultrasound on flux enhancement. Studies are also needed to assess the extra energy consumed with the use of ultrasound and the effect of ultrasound on membrane integrity and structure. In line with this, efforts should also be directed towards fabricating FO membranes that are strong enough to withstand the applied ultrasound frequency.

## 7. Conclusions

The use of ultrasound in FO process can effectively resolve the issue of membrane fouling and concentration polarization and sustain the performance of FO in water desalination. This was determined after a few investigations on the effect of ultrasound on important aspects of FO such as design strategies and fouling phenomenon. Ultrasound assisted FO systems have received limited attention in the literature and thus further investigation in this challenging area is recommended.

## Figures and Tables

**Table 1:** Applications of different configurations of CA/CTA FO membranes in water desalination [39,64] (Permissions No. 5322090836592 and No. License Number 5322091187298).

Material	Membrane Orientation ^a^	Feed Solution	Draw Solution	Water Flux (L/m^2^ h)	Ref.
CA-Flat sheet	AL-FS	NaCl (0.1 M)	Glucose (2 M)	11.6–12.7	[65]
CA-Flat sheet	AL-FS	NaCl	CuSO_4_ (200,000 mg/L)	3.57	[7]
CA-Hollow fiber	AL-FS/AL-DS	DI	MgCl_2_ (2 M) Sucrose (1 M)	17.1 12.9	[66]
CTA–Flat sheet	AL-FS/AL-DS	Seawater (35,000 mg/L NaCl)	Polyacrylic acid sodium salt	18	[67]
Cellulose membrane	AL-DS	DI	4% Polyelectrolyte solution	0.347	[68]
CTA	AL-FS/AL-DS	Brackish water	Na_2_SO_4_	5–13	[10]
CTA	AL-FS/AL-DS	Seawater (0.6 M NaCl)	NaCl, MgCl_2_, CaCl_2_, KCl MgSO_4_, Na_2_SO_4_, C_6_H_12_O	2–25	[11]
CTA spiral wound embedded on a polyester screen	AL-FS	Tap water	Sea salt (99.9% NaCl)	-	[69]
CA-Hollow fiber	AL-FS/AL-DS	DI	MgCl_2_ (2 M)	5.0/7.3	[70]
CA-Flat sheet	AL-FS/AL-DS	DI	MgCl_2_ (2 M)	10.3/17.3	[71]
CA-Hollow fiber	AL-FS/AL-DS	DI	MgCl_2_ (2 M)	8/36	[72]
CA-Hollow fiber	AL-DS	DI	MgCl_2_ (2 M)	17.1	[66]
CTA-Flat sheet	AL-FS/AL-DS	DI	NaCl (2 M)	9.0/12.8	[72]
CA-Flat sheet	AL-FS/AL-DS	DI	NaCl (2 M)	10/13	[73]
CA-Flat sheet	AL-FS/AL-DS	DI	NaCl (2 M)	12/21.6	[62]
CA-Hollow fiber	AL-DS	DI	NaCl (2 M)	17.5	[74]
CA/CTA Flat sheet	AL-FS	DI	NaCl (1 M)	10.39	[75]
CTA-Flat sheet	AL-FS	DI	NaCl (4 M)	28.8	[76]

^a^ AL-FS, active layer facing the feed solution; AL-DS, active layer facing the draw solution.

**Table 2:** Draw solution characteristics and their effect on membrane water flux [14,80] (Permissions No. 5322091417145 and No. 5322100148092).

Characteristic	Level	Effect on the FO Process Performance (Flux)
Osmotic pressure	High	Enhances water flux.
Solubility	High	Enhances water flux (provides high osmotic pressure).
Viscosity	Low	Enhances water flux (provides low resistance).
Diffusivity	High	Enhances water flux.
Molecular weight	Small	Enhances water flux (small molecular weight solutes provide high diffusivity but increase the chance of the reverse solute problem).
Concentration	High	Enhances water flux. However, a very high draw solution concentration increases the concentration polarization effect and thus reduces water flux.
Temperature	High	Enhances water flux. However, high draw solution temperature increases scaling issues and thus reduces the water flux.

**Table 3:** Concentration polarization types based on active layer orientation.

CP Category	Occurrence Location	CP Type	Active Layer Orientation
ECP	Active layer surface	CECP	Feed solution
		DECP	Draw solution
ICP	Within the support layer	DICP	Feed solution
		CICP	Draw solution

**Table 4:** Ultrasound waves frequencies and associated applications [134].

Category	Range	Application
Human hearing	10 Hz–18 kHz	-
Power ultrasound	20 kHz–100 kHz	Cleaning
Extended range	100 kHz–1 MHz	Sonochemistry
High frequency	1 MHz–10 MHz	Medical diagnostics and analysis

## Data Availability

Not applicable.
